# How kidney function adapts to changes in acid–base balance: the AE4 transporter as a central acid–base sensor in tubular cells

**DOI:** 10.1007/s00424-023-02898-6

**Published:** 2023-12-20

**Authors:** Frank Schweda

**Affiliations:** Institute of Physiology, University of Regensburg, Universitaetsstr. 31, 93053 Regensburg, Germany

Maintaining a constant physiological pH is crucial for the normal function of cells and organs, and severe disturbances of acid–base homeostasis can lead to death. In addition to the lungs, which respond rapidly to pH changes by adjusting respiration and exhaling CO_2_, the kidneys are the central organ for maintaining acid–base homeostasis. In keeping with the prominent role of the kidneys, chronic kidney disease (CKD) is accompanied by a disturbed acid–base balance in the form of acidosis. In addition, inherited or acquired dysfunction of certain nephron segments can lead to changes in systemic pH. These disorders are classified into proximal and distal renal tubular acidosis, depending on the nephron segment affected [2, 4]. Distal renal tubular acidosis (dRTA) is caused by disturbances in the distal nephrons including collecting ducts. Here, reduced secretion of protons into the urine can lead to acidosis. In addition to principal cells that are responsible for NaCl and water reabsorption, collecting ducts and upstream connecting tubules contain intercalated cells (IC), which exist in two subtypes: type A and type B. Type A ICs express H^+^-ATPases in their apical membrane (urine-facing) and secrete H^+^, acidifying the urine to a pH of approximately 4.5 to 4 in the case of acidosis. The secreted protons are produced by the hydration of CO_2_, which is mediated by carbonic anhydrase type II. Bicarbonate that is also generated in this reaction is released into the interstitium via a chloride/bicarbonate exchanger, the anion exchanger 1 (AE1) [4]. In contrast to type A ICs, type B ICs release bicarbonate into the urine, for which the apically localized chloride/bicarbonate exchanger pendrin is responsible (Fig. 1) [2, 4]. Pendrin expression and activity are stimulated by alkalosis and suppressed by acidosis. As with type A ICs, bicarbonate is formed by carbonic anhydrase reaction, but in type B ICs, the generated H^+^ is secreted into the interstitium by H^+^-ATPases. This simplified view of renal acid–base regulation is far from complete, and several other transporters and processes have been identified [2, 4]. Despite these findings, key questions regarding the adaptation of renal function to changes in acid–base balance still need to be answered. For example, the question of how ICs detect acidosis or alkalosis in order to adapt their function accordingly has not been clarified. Vitzthum and coworkers have now made a significant advance on this question, as they have identified the AE4 transporter as a central acid–base sensor in type B ICs [3]. AE4 is a Na^+^-dependent chloride/bicarbonate exchanger that is expressed in the basolateral membrane of type B ICs in mice, rats, and humans [3]. To clarify the function of AE4 in ICs, the authors conducted extensive and sophisticated studies using a wide spectrum of methods in AE4 knockout mice (AE4-KO). Based on previous findings suggesting involvement of AE4 in Na^+^-reabsorption and blood volume control [1, 5], the mice were exposed to a low-salt diet. Although there was no evidence of impaired volume control or NaCl reabsorption, AE4-KO had an impaired acid–base status in the form of hypochloremic metabolic alkalosis. This effect was exacerbated by dietary alkali-loading (NaHCO_3_) and a life-threatening hypochloremic alkalosis developed in AE4-KO mice but not in WT mice. As described above, the chloride/bicarbonate exchanger pendrin is responsible for apical secretion of HCO_3_^−^ and reabsorption of Cl^−^ in type B ICs, and its protein abundance is upregulated by alkalosis. While this was the case in WT mice, this adaptation was absent in AE4-KO, and redistribution of pendrin from the subapical cytosolic region to the plasma membrane was also absent. Technically very sophisticated experiments on perfused isolated collecting ducts showed an increased pendrin transport rate in alkalotic WT mice, which was not detectable in alkalotic AE4-KO. Once the role of AE4 in adaptation to metabolic alkalosis was established, AE4-KO mice were exposed to an acid load (NH_4_Cl). Both genotypes developed hyperchloremic acidosis, but the acidosis was more pronounced in the AE4-KO. As expected, downregulation and endocytosis of pendrin from the apical membrane occurred in acidotic WT mice. This physiological adaptation process did not take place in AE4-KO, so they had an increased renal loss of HCO_3_^−^ despite the systemic acidosis. The sensory function of AE4 in type B ICs is therefore not only limited to alkalosis but also plays a role in acidosis. From these and other exciting results, which cannot be discussed here, the authors derive a working model on acid–base sensing in type B ICs: In metabolic alkalosis, AE4 may increase the uptake of HCO_3_^−^ in type B ICs and thereby induce the transcription, translation, and translocation of pendrin into the apical membrane and thus an increased excretion of HCO_3_^−^ into the urine. Conversely, in acidosis, decreased uptake of HCO3^−^ via AE4 leads to reductions of pendrin expression and of urinary HCO_3_^−^ loss (Fig. 1).

**Fig. 1:**
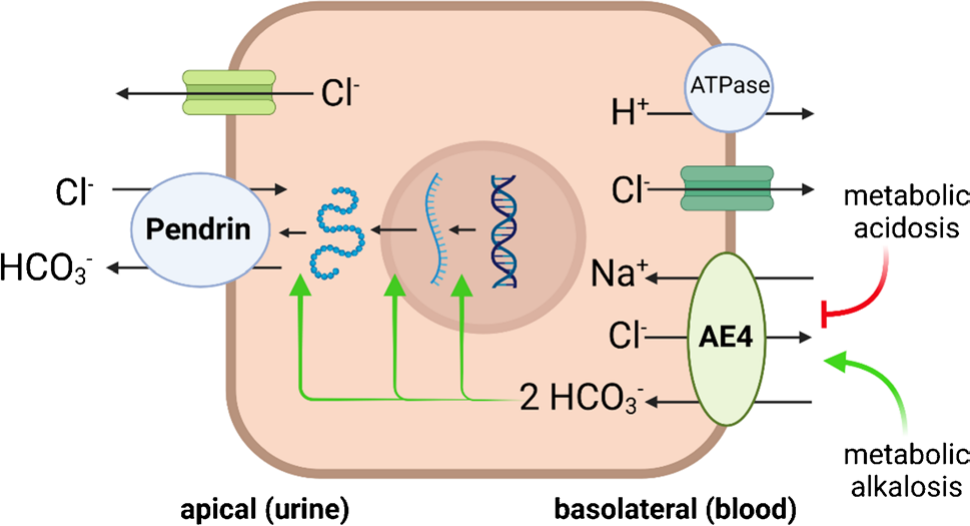
Proposed model for the function of AE4 in type B intercalated cells (adapted from (3))

In metabolic alkalosis, an increased HCO_3_^−^ transport rate of AE4 leads to stimulation of transcription, translation, and translocation of pendrin to the apical membrane and thus to increased HCO_3_^−^ excretion. In metabolic acidosis, the HCO_3_^−^ transport rate of AE4 is reduced, so that pendrin expression and HCO_3_^−^ excretion decrease (Fig. 1 created with BioRender.com).

This very elegant and focused work clearly shows that AE4 plays a vital role in the adaptation of renal function in acid–base disorders and is a sensor of acid–base balance. Furthermore, it opens the door to many follow-up questions, e.g., by which intracellular mechanisms AE4-dependent HCO3^−^ uptake regulates pendrin expression.

## Data Availability

No datasets were generated or analyzed during the current study.
